# Treatment of Angio-Seal® Vascular Closure Device-Induced Acute Femoral Artery Occlusion with SilverHawk® Directional Atherectomy

**DOI:** 10.7759/cureus.910

**Published:** 2016-12-02

**Authors:** Rishi Sharma, Karthik Vamanan, Kamal Gupta

**Affiliations:** 1 Cardiovascular & Renal Research, Kansas City VA Medical Center; 2 Department of Vascular Surgery, Kansas University Hospital, Kansas City; 3 Department of Cardiology, Kansas University Hospital, Kansas City

**Keywords:** angio-seal®, vascular closure device, percutaneous, occlusion, silverhawk®

## Abstract

Vascular closure devices provide a safe and cost-effective method to achieve rapid hemostasis and early ambulation after angiographic procedures. Rarely, they can result in arterial injury with resultant stenosis or acute arterial closure requiring open surgical intervention. We report an Angio-Seal® vascular closure device-induced acute arterial closure successfully treated percutaneously with the SilverHawk® plaque excision system. This report discusses the possible mechanisms of Angio-Seal® induced arterial occlusion and various percutaneous options for treatment.

## Introduction

Vascular closure devices (VCD) are increasingly used in place of manual compression to provide rapid hemostasis and early ambulation after angiographic procedures. They have been shown to reduce hospital stay and have been demonstrated to be cost-effective [1-2]. Although they have equivalent overall safety as compared to manual compression, they can rarely result in arterial injury with resultant stenosis or acute arterial closure [3-4]. This complication has been reported with a variety of such devices and generally needs open surgical intervention. Angio-Seal® (St. Jude Medical, St. Paul, MN) is a commonly used collagen-based VCD. Here, we report an Angio-Seal® VCD-induced complete acute arterial closure treated entirely percutaneously with the SilverHawk® plaque excision system (ev3 Inc., Plymouth, MN). 

## Case presentation

**Figure 1:**
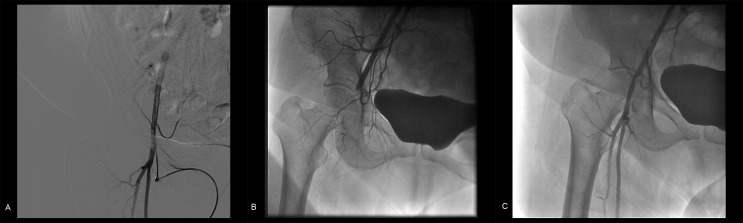
Initial Images A) Initial digital subtraction angiogram of the right common femoral artery showing a normal arterial access site. B) Acute closure of right common femoral artery (CFA) after Angio-Seal deployment. C) Residual 40% stenosis of the right CFA after sequential balloon angioplasty with eccentric stenosis.

A 60-year-old Caucasian female with a history of coronary artery bypass surgery, diabetes mellitus, dyslipidemia, and chronic tobacco use was admitted to the hospital with Rutherford Class III exertional claudication in the bilateral hips and buttocks. Non-invasive workup, including a duplex sonogram, demonstrated distal aortic stenosis involving the aortoiliac bifurcation. She underwent an abdominal aortogram with bilateral lower extremity arterial run-offs. During the procedure, vascular access was obtained with #6 French short sheath, which was advanced into the right common femoral artery without complications. The arteriograms confirmed the presence of a distal aortic 70% calcific stenosis with the plaque involving the ostia of both common iliac arteries. The run-off vessels bilaterally were without significant stenosis. A staged percutaneous procedure was planned. An Angio-Seal® device was deployed successfully after an angiogram confirmed suitability for device placement (Figure 1A). 

Immediately after the Angio-Seal® deployment, the patient developed leg discomfort with the loss of arterial pulses in the right leg. A duplex scan confirmed acute occlusion of the right common femoral artery (CFA). Immediate vascular access was obtained at the contralateral (left) CFA and an angiogram of right CFA confirmed a total occlusion (Figure 1B). A #7 French 45 cm sheath was advanced to the right external iliac artery after crossing the aortoiliac bifurcation from the left side. After some maneuvering, a 0.035-inch Glidewire® (Terumo Med. Corp., Somerset, NJ) could be advanced across the lesion into the right superficial femoral artery (SFA). Sequential angioplasty was performed using 4.0, 6.0, and 8.0 mm balloons. Prolonged low-pressure inflations were performed with the final balloon angioplasty establishing patency of the right CFA with a 40% residual stenosis and a residual eccentric haziness. Distal pulses returned to normal, and thus, the procedure was terminated at that time (Figure 1C).

**Figure 2:**
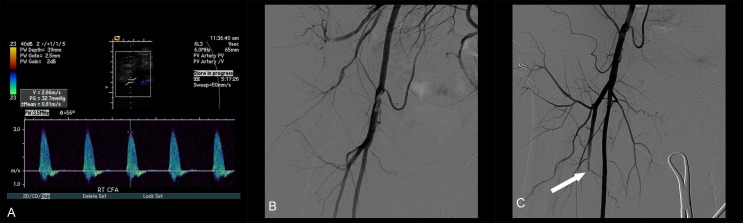
Images After Three Weeks A) Doppler ultrasound of right common femoral artery (CFA) after three weeks of procedure indicating re-stenosis. B) Eccentric hazy lesion with 80% stenosis of right CFA on angiogram. C) Successful treatment of 80% stenotic lesion with SilverHawk atherectomy device with no significant residual stenosis. Distal embolization of profunda femoris artery after atherectomy (arrow).

However, over the next three weeks, she developed worsening claudication in the right lower extremity. Doppler ultrasound showed significantly elevated velocity and turbulence consistent with a hemodynamically significant stenosis at the right common femoral artery (Figure 2A). An angiogram demonstrated an eccentric hazy lesion with a resulting 80% stenosis in the right CFA at the site of the Angio-Seal® deployment (Figure 2B). The lesion appeared to be consistent with an eccentric dissection flap with a superimposed thrombus. After considering open surgical repair, we decided on an initial percutaneous approach and proceeded with a directional atherectomy with the SilverHawk® plaque excision system. In order to prevent distal embolization of excised plaque/dissection flap, we used distal embolic protection using a Spider^TM^ filter device placed at the distal SFA. With multiple passes, the lesion was shaved off resulting in no significant residual stenosis (Figure 2C). The atherectomy material retrieved consisted of a whitish material of intima/media plaque with the intraluminal anchor (foot) of the Angio-Seal® system. A significant amount of debris was also retrieved in the filter basket with some embolization to the profunda femoris artery (Figure 2C arrow). The procedure was otherwise uneventful. She remains asymptomatic at 36 months and repeat Doppler ultrasound showed widely patent artery with no hemodynamically significant stenosis. The distal aortic lesion was subsequently treated percutaneously without incidence.

## Discussion

Angio-Seal® is a collagen-based VCD, which creates a mechanical seal with a bio-absorbable intraluminal polymer anchor with an extraluminal collagen sponge in the tissue track. Hemostasis is achieved primarily by anchor–arteriotomy–collagen sandwich supplemented by coagulation-induced properties of collagen. VCD’s have been rarely associated with acute or subacute occlusion of the arteries. This rare complication was seen between 0.1% and 2.9% [4]. VCD associated arterial stenosis or occlusion may be related to local arterial disease with atheromatous plaques and a routine angiography of the blood vessel is recommended before VCD deployment. This patient did not show any signs of local vascular disease before Angio-Seal® deployment and the duplex scan had been normal at the location of the right CFA.

The possible mechanisms for Angio-Seal® device-induced arterial occlusion include the following: 1) Anchor-induced dissection resulting from the dragging of the anchor along the arterial inner wall during deployment; 2) thrombosis induced by the intraluminal anchor or a portion of the collagen plug that prolapsed into the lumen; and 3) plaque rupture from device-induced trauma, resulting in worsening stenosis at the deployment site. The traditional management for VCD-induced vessel stenosis/occlusion is open surgery and has been well-described [5]. Balloon angioplasty has been used with mixed and often temporary improvement that required subsequent surgical intervention [6]. Such interventions are based on the clinical judgment of the physician and are limited to case reports only.

In the case presented, balloon angioplasty provided temporary relief likely because the local thrombogenic stimulus with anchor plug/ prolapsing collagen plug and associated dissection flap was still present intraluminally. The SilverHawk® atherectomy system was chosen with the thought that the atherectomy would remove the inciting stimulus (the plug or the anchor) as well as also any dissection flap, if present, and minimize the need for a stent in this unfavorable location. Furthermore, a failed atherectomy would likely not compromise a future open surgical repair.

A distal filter device is strongly recommended to prevent the risk of distal embolization with the use of this atherectomy system [7-8]. In the only other such reported case of atherectomy, significant debris was retrieved in the filter and there were no complications [8]. However, as was seen in this current case, these devices are not perfect, and even though the filter basket prevented embolization into the SFA/ infra-genicular arteries, the embolic material did occlude some of the branches of the profunda femoris. Open surgical exploration should be undertaken where the risk of distal embolization outweighs the benefits of SilverHawk® plaque excision system, such as in cases with compromised distal runoff. Another rare but previously described complication of directional atherectomy is a pseudo-aneurysm formation that may require subsequent surgical repair [9-10].

## Conclusions

The use of an Angio-Seal® VCD may rarely result in an acute closure of the artery. Such a stenosis/occlusion may not be treated successfully with angioplasty alone. Although surgical intervention has been the traditional treatment, SilverHawk® plaque excision system can be used successfully and, thus, avoid open surgical repair. However, the use of a distal protection device is strongly recommended. 
